# Grape Seed Extract to Improve Liver Function in Patients with Nonalcoholic Fatty Liver Change

**DOI:** 10.4103/1319-3767.65197

**Published:** 2010-07

**Authors:** Manouchehr Khoshbaten, Akbar Aliasgarzadeh, Koorosh Masnadi, Sara Farhang, Mohammad K. Tarzamani, Hosain Babaei, Javad Kiani, Maryam Zaare, Farzad Najafipoor

**Affiliations:** Department of Drug Applied Research Center, Tabriz University of Medical Sciences, Iran

**Keywords:** Grape seed, treatment, liver function tests, liver hemodynamics, nonalcoholic fatty liver disease, treatment

## Abstract

**Background/Aim:**

Therapeutic interventions in nonalcoholic fatty liver disease are limited, while antioxidative materials have shown benefi ts in animal models. This study aimed to evaluate grape seed extract as an anti-oxidative material in this process. Therapeutic effects of grape seed extract were evaluated in comparison to vitamin C in a double-blind setting.

**Materials and Methods:**

Fifteen patients were enrolled in each group. Liver function tests were done; also, grade of steatosis and pattern of echogenicity of the liver were determined. Patients were followed up by the same evaluation repeated in first, second and third months.

**Results:**

Mean age ± standard deviation was 43.2 ± 10.3 years. Grape seed extract (GSE) significantly improved the grade of fatty liver change; and resulted in significant decrease in alanine aminotransferase in patients receiving the concentrate compared to those receiving vitamin C independently, from the initial grade of steatosis.

**Conclusions:**

This study describes the beneficial effect of using grape seed extract for three months in patients with nonalcoholic fatty liver disease. These results may improve with a longer period of follow-up.

Nonalcoholic fatty liver disease (NAFLD) is a progressively more documented condition. Nonalcoholic fatty liver disease affects 10 - 24% of the general population in various countries and may progress to end-stage liver disease.[1] Mildly to moderately elevated serum levels of aspartate aminotransferase (AST), alanine aminotransferase (ALT) or both are the most common and often the only laboratory abnormality found in patients with NAFLD.[2]

The wide spectrum of NAFLD is an expression of various histopathologic features, including steatosis, mixed inflammatory-cell infiltration, hepatocyte ballooning and necrosis, glycogen nuclei, Mallory's hyaline and fibrosis. Increased intrahepatic levels of fatty acids provide a source of oxidative stress, which may, in large part, be responsible for the progression from steatosis to steatohepatitis to cirrhosis. No medications have been standardized for NAFLD, and clinical trials are ongoing; free-radical scavengers and antioxidant agents are thought to be useful.

There is no therapy for NAFLD that has been proven to be clearly effective. Anti-oxidative materials have shown protective effects on liver injury in animals. Vitamins C and E combination therapy has been investigated in NAFLD in some studies, which revealed improvements in fibrosis and ALT levels with this therapy.[3,4]

Grape seed extract (GSE) has been found to reduce the severity of ischemic/reperfusion-induced organ injury through its ability to balance the oxidant-antioxidant status, to inhibit neutrophil infiltration and to regulate the release of inflammatory mediators.[5] A polyphenol contained in grape seeds is resveratrol, which may interfere with cancer cell growth and proliferation, as well as induce apoptosis.[6,7] In this study, the significance of grape seed extract treatment in adult patients with NAFLD was investigated in comparison to treatment with vitamin C.

## MATERIALS AND METHODS

The study was arranged at clinics of Tabriz University of Medical Sciences during 2008, and protocols were approved by the Ethical Committee of Tabriz University of Medical Sciences. All the patients were recruited from the university clinic. They were mostly referred for more assessment of elevated levels of liver enzymes or had visited the clinic for the first time with nonspecific complaints like abdominal discomfort. All patients gave written consent to join this trial.

The diagnosis of NAFLD was based on clinical examinations, elevated level of liver enzymes, evaluation of the liver by ultrasonography and excluding other etiologies for fatty liver. None of the patients had any malignancy or inflammatory disease.

A detailed history was taken and patients with history of alcohol consumption or use of medications known to precipitate steatohepatitis, lipid-reducing agents, ursodeoxycholic acid or vitamin supplements in the six months prior to the study were excluded. Laboratory evaluation in this study included serum liver tests for AST, ALT and alkaline transaminase (ALT) and alkaline phosphatase (ALP). Further investigations included a hepatobiliary system ultrasound, viral serology, autoantibody titers, serum iron, ferritin and transferrin saturation, ceruloplasmin and urine copper levels. All patients were negative for hepatitis B serological tests, antibody to hepatitis C virus and autoantibodies (antinuclear antibody [ANA], anti-mitochonfrial antibody [AMA], anti-smooth muscle antibody [ASMA] and anti-LKM). Serum electrolytes, urea, creatinine, fasting glucose, complete blood count, cholesterol and triglyceride levels were also obtained. Iron profile, a1-antitripsine and serum ceruloplasmin levels were normal in all patients.

Then, patients with continuous NAFLD were included in this study. They were given a code name and were referred to receive the medication randomly. They received either GSE or vitamin C (1000 mg per 12 hours, Zahravi Pharm. Co., Iran) randomly. Serum biochemistry and ultrasonographic measurement of liver and spleen were performed at entry and every month.

Crushed grape seeds (Vitis vinifera) were extracted in 95% ethanol with mechanical agitation for 2 to 3 hours, and this process was repeated twice. The organic solvent was then evaporated, and the crude extract was partitioned between H_2_O and n-hexane to separate lipoid compounds.

The aqueous solution was evaporated to dryness using rotary evaporator (40°C) to yield approximately 2.6 g of extract/100 g of seeds and was filled in 100 mg capsules to be used orally in the present study.[8] Moreover, all of the patients received advices for necessary modifications of life style, particularly to do exercises, take appropriate diet or additional medications when necessary.

All data were expressed as mean ± SD (standard deviation). Characteristics of the two groups of patients were compared by Chi-square and Student t test as appropriate. A repeated-measures ANOVA was used to compare the data before and after each treatment and therapeutic results of each supplement. A P value of 0.05 was considered statistically significant.

## RESULTS

Fifteen patients could successfully complete the process in each group, including regular follow-up. The GSE group included 10 males and 5 females. Mean (SD) age of these patients was 39.9 (9.4) years. Five (33.3%) patients were in the range of healthy weight (< 24.9), 6 (40%) were overweight and 4 (26.7%) were obese. During the initial diagnosis, 2 patients were found to have diabetes mellitus, 2 were with hypertension and 3 were with hyperlipidemia, in all of whom diet and life style modifications were recommended as the first line of treatment.

Ten females and 5 males participated as controls; they received vitamin C. Mean age ± SD of the controls was 46.8 ± 10.4 years. The control group had no significant difference in terms of age (*P* = 0.084) and gender (*P* = 0.068) in comparison with GSE group. The two groups were also matched with regard to the number of patients with diabetes mellitus (*n* = 2, *P* = 0.109), hypertension (*n* = 1, *P* = 1.000) and hyperlipidemia (*n* = 2, *P* = 0.598).

**Figure 1:**
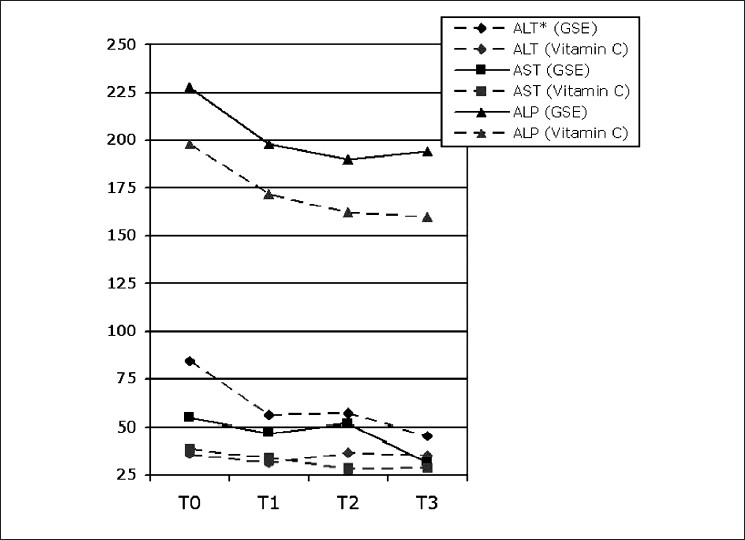
Estimated marginal means of serum liver test results in patients receiving grape seed extract for three months (AST: aspartate transaminase, ALT: alanine transaminase, ALP: alkaline phosphatase. *: The decrease reaches statistical significance)

Results of serum liver tests in patients receiving GSE are listed in Table 1, containing details of initial diagnosis and repeated evaluations at one-month intervals. There was no statistically significant difference in the levels of AST and ALP during these three months in both the groups (*P*= 0.760 and *P*= 0.977, respectively). However, serum level of ALT decreased significantly in patients treated with GSE when compared to controls (*P* = 0.012). Figure 1 shows more precisely the estimated marginal means of these results in both groups, in which the decrease in ALT reached the level of significance. This effect was independent from the pattern of the liver reported by ultrasonography in initial diagnosis.

Sizes of the liver, the spleen and portal vein were also evaluated. The results are listed in Table 1. No significant change was observed in the size of the liver (*P* = 0.440), the spleen (*P* = 0.366) and the portal vein (*P* = 0.490) in the two groups.

**Table 1:** Serum liver tests and hemodynamic parameters in patients with nonalcoholic fatty liver disease receiving grape seed extract, measured at one-month intervals as mean ± SD

	T0	T1	T2	T3
ALT	84.3 ± 22.1	56.4 ± 36.8	57.3 ± 30.6	45.5 ± 22.1
AST	60.4 ± 25.2	47.0 ± 29.8	51.7 ± 37.5	31.7 ± 8.1
ALP	227.7 ± 81.9	198.0 ± 73.0	190.0 ± 61.0	194.0 ± 95.6
Liver span	170.0 ± 27.6	159.0 ± 25.3	158.3 ± 13.3	156.6 ± 14.5
Spleen span	116.0 ± 25.6	131.5 ± 40.9	110.5 ± 55.6	122.0 ± 36.2
Portal vein diameter	9.0 ± 3.5	9.0 ± 3.5	9.8 ± 1.2	9.7 ± 1.6

AST: Aspartate transaminase, ALT: Alanine transaminase, ALP: Alkaline phosphatase

Additionally, GSE significantly improved grade of steatosis in our study population (*P* < 0.001), which is described in Table 2. Similar outcome was not observed by using vitamin C. No adverse drug effects were reported by patients during the study.

**Table 2:** Grade of liver steatosis reported by ultrasonographic evaluation in patients with nonalcoholic fatty liver disease receiving grape seed extract, measured at one-month intervals

	T0	T1	T2	T3
Normal	0	1 (6.7)	2 (13.3)	4 (26.7)
Grade 1	8 (53.7)	12 (80.0)	13 (86.7)	11 (73.3)
Grade 2	7 (46.7)	2 (13.3)	0	0

## DISCUSSION

The present study was undertaken to assess the effect of a three-month dietary supplementation with GSE on biological function of the liver, as well as hemodynamic status. Another well-matched group receiving vitamin C was defined as control. To our knowledge, only a few studies have been carried out to examine the effect of GSE on liver function in patients with NAFLD, and in particular, on liver hemodynamics. We described the effectiveness of three-month consumption of GSE in decreasing ALT, as well as a significant improvement in the grade of fatty liver change.

NAFLD represents a spectrum of liver diseases characterized mainly by macrovesicular steatosis in the absence of significant alcohol ingestion. NAFLD can be a precursor for nonalcoholic steatohepatitis (NASH), which has been found to lead to progressive fibrosis and cirrhosis. Oxidative stress plays an essential role by causing peroxidation of lipids in the hepatocyte membrane to initiate liver fibrosis. Lipid peroxidation and the generation of free radicals can result in cellular death and hepatic necrosis[9] and contribute to impaired cellular function.[10] Antioxidant supplements could potentially protect cellular structures against oxidative stress. Thus far, therapeutic interventions in NAFLD and NASH are limited.

Until now, there have been few studies which used GSE for the treatment of oxidative stresses, but yet there is no consensus about the therapeutic benefits.[11] Literature reveals anti-oxidative potential of GSE in animal models.[12] The present study is the first one to evaluate therapeutic effect of GSE in patients with NAFLD.

The antioxidant effect has been described for GSE proanthocyanidins in diabetic rats and has been shown to lead to a decrease in the oxidant generation and lipid peroxidation.[13] Also, a protective effect of GSE has been reported on reperfusion-induced injury in rats.[5] GSE could reverse ALT, AST and histological alterations induced by the injury. The therapeutic effect of GSE was established against bile duct ligation-induced hepatic fibrosis, where oxidative stress takes place; while a 28-day administration of 50 mg/day of GSE successfully decreased ALT and AST after the damage.[14]

Clinical features of NASH are very similar to those of NAFLD, but there are no noninvasive tools to definitely distinguish between steatohepatitis and simple steatosis. The current study is limited by the absence of liver histological findings (inability to perform liver biopsy on ethical grounds), serving as a confirmation of the diagnosis, and is thus restricted to patients diagnosed incidentally to have liver steatosis by ultrasonographic assessments. However; the study population had special characteristics. They were patients with NAFLD who had elevated liver enzymes. Such a condition is associated with a clinically significant risk of developing end-stage liver disease.[15]

The therapeutic effect of GSE on the patients in this study was limited to the decrease in the serum level of ALT, which seems to be the most important parameter representing liver function. The relationship between ALT and NAFLD is not explained completely, but studies report ALT as not only a consequence but also a predictor of developing NASH.[16] Evaluation of liver specimens after the intervention could have explained the results of the current study, but facility for such evaluation was not available in the present setup.

The other finding of the current study was a significant downgrade in steatosis caused by the use of GSE, which was not achieved by using vitamin C. The studies about successful life style interventions to treat fatty liver changes demonstrate a decrease in the level of steatosis, along with improvement in the liver profile. None of the previous studies have evaluated possible effects of antioxidants on ultrasonographic features of the liver; however, these are described in the present study. While it is not clear which of the two (liver biochemical parameters or histological steatosis) begins to improve first, a longer duration of treatment with GSE may render better results. The beneficial effect of using GSE has been described in this study, which may favor consideration of the use of GSE, along with life style modifications.[17]

## References

[1] Angulo P (2002). Nonalcoholic fatty liver disease. N Engl J Med.

[2] Angulo P, Keach JC, Batts KP, Lindor KD (1999). Independent predictors of liver fibrosis in patients with nonalcoholic steatohepatitis. Hepatology.

[3] Harrison SA, Torgerson S, Hayashi P, Ward J, Schenker S (2003). Vitamin E and vitamin C treatment improves fibrosis in patients with nonalcoholic steatohepatitis. Am J Gastroenterol.

[4] Patrick L (2002). Nonalcohoic fatty liver disease: Relationship to insulin sensitivity and oxidative stress: Treatment approaches using vitamin E, magnesium and betaine. Altern Med Rev.

[5] Sehirli O, Ozel Y, Dulundu E, Topaloglu U, Ercan F, Sener G (2008). Grape seed extract treatment reduces hepatic ischemia-reperfusion injury in rats. Phytother Res.

[6] Bagchi D, Garg A, Krohn RL, Bagchi M, Tran MX, Stohs SJ (1997). Oxygen free radical scavenging abilities of vitamins C and E, and a grape seed proanthocyanidin extract *in vitro*. Res Commun Mol Pathol Pharmacol.

[7] Kundu JK, Surh YJ (2008). Cancer chemopreventive and therapeutic potential of resveratrol: Mechanistic perspectives. Cancer Lett.

[8] Ray S, Bagchi D, Lim PM, Bagchi M, Gross SM, Kothari SC (2001). Acute and long-term safety evaluation of a novel IH636 grape seed proanthocyanidin extract. Res Commun Mol Pathol Pharmacol.

[9] Chitturi S, Farrell GC (2001). Etiopathogenesis of non-alcoholic steatohepatitis. Semin Liver Dis.

[10] Stark G (2005). Functional consequences of oxidative membrane damage. J Membr Biol.

[11] Lirussi F, Azzalini L, Orando S, Orlando R, Angelico F (2007). Antioxidant supplements for non-alcoholic fatty liver disease and/or steatohepatitis. Cochrane Database Syst Rev.

[12] Morin B, Narbonne JF, Ribera D, Badouard C, Ravanat JL (2008). Effect of dietary fat-soluble vitamins A and E and proanthocyanidin-rich extract from grape seeds on oxidative DNA damage in rats. Food Chem Toxicol.

[13] El-Alfy AT, Ahmed AA, Fatani AJ (2005). Protective effect of red grape seeds proanthocyanidins against induction of diabetes by alloxan in rats. Pharmacol Res.

[14] Dulundu E, Ozel Y, Topaloglu U, Toklu H, Ercan F, Gedik N (2007). Grape seed extract reduces oxidative stress and fibrosis in experimental biliary obstruction. J Gastroenterol Hepatol.

[15] Ekstedt M, Franzén LE, Mathiesen UL, Thorelius L, Holmqvist M, Bodemar G (2006). Long-term follow-up of patients with NAFLD and elevated liver enzymes Hepatology.

[16] Schindhelm RK, Diamant M, Dekker JM, Tushuizen ME, Teerlink T, Heine RJ (2006). Alanine aminotransferase as a marker of non-alcoholic fatty liver disease in relation to type 2 diabetes mellitus and cardiovascular disease. Diabetes Metab Res Rev.

[17] Ueno T, Sugawara H, Sujaku K, Hashimoto O, Tsuji R, Tamaki S (1997). Therapeutic effects of restricted diet and exercise in obese patients with fatty liver. J Hepatol.

